# The effectiveness of inhaled *Cannabis* flower for the treatment of agitation/irritability, anxiety, and common stress

**DOI:** 10.1186/s42238-020-00051-z

**Published:** 2020-12-09

**Authors:** Sarah S. Stith, Xiaoxue Li, Jegason P. Diviant, Franco C. Brockelman, Keenan S. Keeling, Branden Hall, Jacob M. Vigil

**Affiliations:** 1 Department of Economics, University of New Mexico, Albuquerque, USA; 2 Department of Psychology, University of New Mexico, Albuquerque, USA; 3 MoreBetter, Ltd., Washington, USA

**Keywords:** Anxiety, *Cannabis*, Marijuana, Cannabidiol, Tetrahydrocannabinol, Stress, Negative affect

## Abstract

**Background:**

An observational research design was used to evaluate which types of commonly labeled *Cannabis* flower product characteristics are associated with changes in momentary feelings of distress-related symptoms.

**Methods:**

We used data from 2306 patient-directed cannabis administration sessions among 670 people who used the real-time *Cannabis* effects recording software, Releaf App, between June 6, 2016, and February 23, 2019, for tracking the effects of *Cannabis* flower consumption. Fixed effects multivariable panel regression techniques were used to establish overall relief by symptom type and to determine which labeled product characteristics (e.g., subspecies/subtype, inhalation method, and major cannabinoid contents) showed the strongest correlation with changes in momentary feelings of agitation/irritability, anxiety, and stress, along with experienced side effects.

**Results:**

In total, a decrease in symptom intensity levels was reported in 95.51% of *Cannabis* usage sessions, an increase in 2.32% of sessions, and no change in 2.16% of sessions. Fixed effects models showed, on average, respondents recorded a maximum symptom intensity reduction of 4.33 points for agitation/irritability (SE = 0.20, *p* < 0.01), 3.47 points for anxiety (SE = 0.13, *p* < 0.01), and 3.98 for stress (SE = 0.12, *p* < 0.01) on an 11-point visual analog scale. Fixed effects regressions showed that, controlling for time-invariant user characteristics, mid and high tetrahydrocannabinol (THC) levels were the primary independent predictor of increased symptom relief, and that when broken out by symptom type, this effect was only statistically significant for our largest sample of users, those reporting anxiety rather than agitation/irritability or stress. Cannabidiol (CBD) levels were generally not associated with changes in symptom intensity levels. In a minority of cannabis use sessions (< 13%), cannabis users reported anxiogenic-related negative side effects (e.g., feeling anxious, irritable, paranoid, rapid pulse, or restless), whereas in a majority of sessions (about 66%), users reported positive anxiolytic side effects (e.g., feeling chill, comfy, happy, optimistic, peaceful, or relaxed).

**Conclusions:**

The findings suggest the majority of patients in our sample experienced relief from distress-related symptoms following consumption of *Cannabis* flower, and that among product characteristics, higher THC levels were the strongest predictors of relief.

**Supplementary Information:**

The online version contains supplementary material available at 10.1186/s42238-020-00051-z.

## Background

Americans experience some of the highest levels of stress in the world (Gallup 2019), with over 50% of recently surveyed adults reporting concerns over issues such as “The future of our nation” (63%), “money” (62%), “work” (61%), “current political environment” (57%), and “violence and crime” (51%) according to the American Psychological Association (2017). Stress and anxiety are also among the most common health symptoms for which pharmaceutical medications are prescribed—often for extended periods of time—and are core features of numerous mental and physical health conditions, including depression, addiction, eating disorders, schizophrenia, autism, attention-deficit/hyperactivity disorder, and acute and chronic physical illness and pain (Bandelow et al. 2017; Gureje 2008; De Heer et al. 2014). The most commonly prescribed pharmaceutical medications for symptoms of anxiety include sedatives (e.g., benzodiazepines), antidepressants (SSRIs, SNRIs), antihistamines, and anticonvulsant medicines, with many people also seeking relief through the use of alcohol and illicit drugs (Bandelow and Michaelis 2015; Man et al. 2015; Slee et al. 2019). Alcohol and many conventional psychiatric medications are associated with frequent and severe negative side effects (e.g., addiction and suicidality), adverse reactions, acute toxicity, and even risk of death (Dodds 2017; Kurlawala et al. 2018; Muller-Oerlinghausen and Berghofer 1999; Read and Williams 2018; Wick 2013).

Stress- and anxiety-related health conditions, particularly post-traumatic stress disorder (PTSD) and chronic pain, are also among the most common health conditions among patients enrolled in state-authorized medical cannabis programs throughout the United States (U.S.) and reasons why people report using and substituting the *Cannabis* plant for several major classes of medications (e.g., opiates, sedatives, antidepressants) more generally (Piper et al. 2017; Stith et al. 2018a; Stith et al. 2018b; Vigil et al. 2017). According to National Academies of Sciences, E. and M (2017) Committee on the Health Effects of Marijuana, there remains limited clinical evidence that cannabis products offer effective treatment for the improvement of anxiety symptoms, while also acknowledging the scarcity of information regarding routes of administration, dose, efficacy, or side effects of common, commercially available cannabis products in the U.S. This lack of information arises primarily from historical federal regulatory barriers to assessing the *Cannabis* plant’s medicinal potential, which have largely limited investigations to cannabis-derived formulates or synthetic analog therapies not widely generalizable to the vast range of common, commercially available products used by millions of people every day (National Academies of Sciences, E. and M 2017; Stith and Vigil 2016). Few studies to date attempt to measure how the broad range of cannabis products, with widely varying cannabinoid contents and ingestion methods, affect momentary symptoms of distress under naturalistic circumstances (Cuttler et al. 2018; Stith et al. 2019; Stith et al. 2018b).

Animal model studies suggest that some of the major cannabinoids (namely cannabidiol (CBD)) have dose-dependent biphasic effects (Andrade et al. 2019), exhibiting anxiolytic and antidepressant effects at lower doses (Schier et al. 2014) and anxiogenic responses at higher doses (Kasten et al. 2019). In humans, frequent cannabis use is correlated with higher rates of anxiety disorders, though the direction of causality remains elusive (Crippa et al. 2009; Shalit and Lev-Ran 2020). Retrospective survey data suggests that CBD in particular may be effective for reducing social anxiety and core symptoms of post-traumatic stress disorder (Bonaccorso et al. 2019; Orsolini et al. 2019; Sarris et al. 2020; Van Ameringen et al. 2020). However, there is also increasing interest in the therapeutic value of capitalizing on the synergistic potential of multiple cannabinoids, terpenes, and flavonoids, or what is often described as the “entourage effect” for treatment of anxiety and other mood disorders (Ferber et al. 2019; Russo 2011). Few studies have sought to measure how consumption of *Cannabis* flower, the most prevalent type of product used in the U.S. (Stith et al. 2019), affects momentary distress-related symptom levels in real time, along with side effect experiences (e.g., paranoia versus relaxation) that may also contribute to *Cannabis*’ potential anxiogenic or anxiolytic effects.

We analyze one of the largest databases of cannabis user-reported real-time administration sessions in the U.S. for measuring which types of *Cannabis* flower product characteristics are associated with momentary feelings of distress-related symptom intensity levels and side effect manifestation, taking into account the wide range of characteristics of flower products from cannabinoid content to inhalation method. This research question was operationalized using the mobile software application (app), Releaf App (2019), which was designed for patients to record the types of products, cannabis subtypes or subspecies, cannabinoid contents, consumption methods, and changes in symptom intensity levels and experienced side effects following cannabis consumption, in real time. (Unlike other similar apps, the Releaf App does not incentivize users to enter sessions by rewarding them through earning points towards free products or other forms of compensation.) A previous study using app-based electronically recorded data found that cannabis users report significant reductions in stress following consumption of inhalable cannabis products (e.g., concentrates, oils, and flower) with higher THC and CBD levels (Cuttler et al. 2018). However, products such as concentrates (e.g., dabs) often differ from dried natural flower in their representative constituents (e.g., cannabinoid, terpene, and flavonoid contents) and additives (e.g., solvents), and in this previous study, it was unclear how different types of cannabis products affected users (Cuttler et al. 2018). Recent findings have also suggested that the increasing THC can have opposite effects depending on the baseline symptom intensity (Childs et al. 2017).

Rather than including a wide range of formulated and natural cannabis products and treating THC and CBD potency levels (%/dry wt.) only as continuous measures, we focused exclusively on *Cannabis* flower and allowed the effects of THC and CBD to vary both linearly and nonlinearly (e.g., low, medium, and high), while controlling for baseline symptom intensity, given that individuals with higher baseline symptom levels have a greater potential for symptom relief, while individuals with lower baseline symptom levels have a greater potential for symptom exacerbation. Furthermore, we accounted for type of strain as marketed (hybrid, sativa, indica), inhalation method (joint, pipe, and vape), session length, and time-invariant user characteristics. This research design enabled us to address the question of which types of commonly labeled *Cannabis* flower characteristics—within the restricted number of potency level options in which *Cannabis* flower “strains” are typically marketed (low, medium, high)—affect changes in distress-related symptom severity. In the current study, people who consumed *Cannabis* flower for treatment of one of three possible types of distressful (negative affect–related) symptom categories, colloquially phrased “agitation/irritability,” “anxiety,” or “stress,” reported symptom intensity levels immediately prior to and following normative *Cannabis* consumption and side effects experienced under typical naturalistic circumstances.

## Methods

### Study design

The study design qualified for exempt status by the University of New Mexico Institutional Review Board, because it posed minimal risk to participants. The Releaf App Privacy Policy, to which users must consent before beginning use, clearly states that anonymized data may be made available to outside researchers. Observational de-identified data subject to an investigator confidentiality agreement were obtained through the owner of the Releaf App™, MoreBetter, Ltd. The Releaf App is a publicly available educational software application that is free to download, compatible with both iOS and Android operating systems, and can easily be found through searching the Internet or via websites like cnet.com. In addition, some dispensaries encourage their customers to use the app to help them identify the best cannabis products for their condition. In general, the app is promoted through word of mouth rather than via paid advertising. Real-time session-level effects were recorded by users of the Releaf App. Patients are prompted to indicate their health condition symptom intensity levels on a 0–10-point visual analog scale, the information that is labeled on the cannabis products they are consuming, and symptom levels and side effects experienced immediately following consumption (Stith et al. 2019; Stith et al. 2018b). More specifically, the app guides the users through a series of screens, first directing users to “select a symptom,” then “select cannabis” (i.e., specific product used) and “select equipment” (e.g., joint, pipe, vape), before directing the user to “set symptom level.” Once the initial symptom intensity is entered, users can update the symptom level at any time before the end of the session. During an active session, users may also enter optional side effects in response to the questions “How does your mind feel?,” “How does your body feel?,” “How’s your mood?,” and “Any other side effects?” before ending and rating the session.

The app includes 50 negative symptoms along with “wellness” that the user can select as the target of their cannabis treatment, with the user capable of treating more than one symptom simultaneously in a session. Out of these 51 options, we selected the three distress-related symptoms available for selection in the app: agitation/irritability, anxiety, and stress. The app also includes 47 side effects (called “feelings” in the user interface), which the user can report at any time during a session. The available symptoms and side effects were generated through focus groups, by the app developers, and by beta user suggestion. Sessions where patients treated a distress-related symptom were included. Only sessions with baseline symptom intensity levels exceeding zero were included in order to allow for the existence of a treatment effect. We further restricted our sample to symptom levels reported within 4 h post-cannabis consumption, similar to previous investigations (Cuttler et al. 2018; Vigil et al. 2018). In other words, we included only sessions with at least one post-cannabis symptom level reported within 4 h. A total of 23,055 cannabis administration sessions, recorded by 4127 individuals, reported a baseline symptom intensity of one or greater for at least one cannabis administration session used to treat anxiety, agitation/irritability, or stress. We further restricted the sample to include only sessions that reported inhaling dried, natural flower, the most common and homogenous type of cannabis product recorded in the Releaf App data (Stith et al. 2019), leaving 14,693 sessions recorded by 3061 users. Because THC and CBD levels are not mandatory recording, these variables are less commonly reported, and our sample is, therefore, further reduced when we restrict the sample to cannabis administration sessions with a full set of product characteristics (subspecies, inhalation method, and THC and CBD levels) reported. We also did not include sessions with THC or CBD levels exceeding 30%/dry wt. because levels exceeding 30% are unlikely to occur naturally in the *Cannabis* plant. Our THC and CBD measures are not mutually exclusive product categories, but rather track potencies, from 0 to 100%, as voluntarily reported by users, presumably based on product labeling. (Including only sessions with THC and CBD reported potentially biases our sample towards sessions using products purchased from dispensaries. All recreational and medical retail markets in the U.S. require labeled independent potency testing by certified laboratories, but individuals, who may, for example, be home cultivating, are unlikely to have access to the necessary equipment or be willing to pay prices designed for commercial retailers testing large product batches.) The final analysis sample includes 2306 cannabis administration sessions by 670 individuals who recorded at least one user session between June 06, 2016, and February 23, 2019. Among these sessions, 18.3% reported agitation/irritability, 43.3% reported anxiety, and 38.4% reported stress. Side effect reporting is optional, so our side effect analysis is restricted to a sample of 1519 sessions recorded by 559 users.

### Study outcomes

The study outcomes are the change in symptom severity level (symptom relief) and the prevalence of side effects following cannabis consumption. Symptom relief is measured as the minimum symptom severity level within 4 h minus the baseline symptom intensity. All cannabis sessions in our final sample include at least one symptom update within 4 h following cannabis consumption with 2.6 (SD = 1.8) symptom updates in the average session. The resulting symptom relief outcome ranges between − 10 (maximum relief) and 9 (maximum exacerbation). In addition to our primary outcome, maximum symptom relief, we also report results for symptom relief within the specified time periods of 1, 2, 3, and 4 h, i.e., the last symptom level reported within that time period minus the baseline symptom intensity. To measure the prevalence of side effects, we used dummy variables to indicate if the user reported any of the side effects in the category as well as variables measuring the proportion of total side effects selected by the user within each category.

### Statistical analysis

A multivariable panel regression approach was used to analyze the association between symptom intensity level and cannabis use and the association between product characteristics and symptom relief, controlling for baseline symptom intensity and session length (minutes). To address the concern that symptom intensity changes in response to cannabis reported by the same user are systematically correlated due to individual-specific characteristics, user-specific fixed effects models were used to account for time-invariant user-specific attributes. As such, the effect of cannabis use on symptom intensity level was estimated from a comparison of symptom intensity levels reported by the same user before and after cannabis use. Similarly, the effect of product characteristics on symptom relief was estimated from a comparison across different products by the same user, rather than a comparison across users.

To examine the average effect of cannabis on symptom intensity by symptom type, we regressed symptom intensity levels on a dummy variable equal to one if symptom intensity was reported after cannabis use and equal to zero if reported before cannabis use, controlling for individual fixed effects and running the regressions separately by symptom type.

To explore the effect of product characteristics on symptom relief, we regressed symptom relief on the product characteristics, including THC and CBD content, labeled subtype (hybrid, *C. indica*, or *C. sativa*), and inhalation method (joint, pipe, and vaporizer). Our primary THC and CBD measures are the potency from 0 to 30%/dry wt. Our plant subspecies variables distinguish between *C. indica*, *C. sativa*, and hybrid *Cannabis* strains. While the colloquial distinction between *C. indica* and *C. sativa* has been widely discounted by the scientific community (Piomelli and Russo 2016), we included these labels because they are still commonly incorporated into *Cannabis* consumer purchasing decisions. For example, Ontario’s government-run online cannabis store differentiates between sativa- and indica-dominant strains as does Leafly, the largest aggregator of consumer-friendly cannabis information in the world with more than 100 million visitors each year. We include inhalation method (joint, pipe, or vaporizer) because joints typically are thought to contain lower quality cannabis than loose flower and vaporizing can occur at lower temperatures than combustion via joint or pipe, making controlling for these characteristics potentially important. Our regressions are run for the overall sample and for the three subsamples defined by symptom type. In addition to including product characteristics, we also controlled for session-level pre-cannabis use symptom intensity and session length (minutes up to 4 h—symptom updates beyond 4 h are not included in our analysis). Baseline symptom intensity is included in all regressions because higher starting symptom levels are associated with greater symptom relief (Vigil et al. 2018). Session length (time from start until the last symptom was reported within 4 h) is included because the effects of inhaled cannabis may vary systematically with session length. Throughout our regression analyses, standard errors were clustered at the user level and to control for heteroskedasticity and arbitrary correlation among sessions entered by the same user.

In addition to our continuous THC and CBD potency measures, we further explore the relationship between THC, CBD, and symptom relief using categorical THC and CBD measures to capture nonlinearities in the effect of THC and CBD on symptom relief. We divided our sample fairly evenly into low THC = < 9%, medium THC = 10–19%, and high THC = 20–30%; and low CBD = 0%, medium THC = 1–9%, and high CBD = 10–30%.

Because we find THC to be a primary driver of symptom relief in the results and it might vary with the other product characteristics, we also test for whether plant subspecies or inhalation method influences the effect of THC on symptom relief, by interacting our continuous measure of THC with those product characteristics. A statistically significant interaction term could arise if, for example, vaporization of cannabis occurs at lower temperatures than combustion of flower in a pipe or joint and this affects THC bioavailability or if joints systematically contain lower grade flower, in which, for example, a greater amount of THC may have already degraded into CBN (cannabinol). We also interact THC with session length to test for variation in the effect of THC over time within 4 h.

We conduct two robustness checks on our symptom relief regression approach. First, because our regression design is inherently based on repeated sessions entered by the same user, we test the robustness of our main results to including only users who entered at least three, four, or five sessions respectively. Second, we extend our time-to-effect analysis by exchanging the maximum symptom relief reported within 4 h for the difference between baseline symptom intensity and the last symptom level reported within 1, 2, 3, and 4 h.

For the side effect outcomes, we use the same regression approach, including the three categories of product characteristics (subtype, inhalation method, and cannabinoid content), baseline symptom intensity level, and session length, along with user fixed effects.

All statistical analyses are conducted using Stata 15.1 (Stata corporation, U.S.).

## Results

Overall, users experienced a symptom intensity reduction in 95.51% of sessions, no change in symptom intensity in 2.16% of sessions, and increases in symptom intensity in 2.32% of sessions. Table 1 presents descriptive statistics for the product characteristics, the starting and minimum symptom severity levels, and the prevalence of side effects. The average cannabis use session involved vaporizing a hybrid strain with at least 10%/dry wt. THC and 1%/dry wt. CBD. On average, baseline symptom intensity levels were 5.45 (SD = 2.14) and minimum post-cannabis symptom intensities were 1.63 (SD = 1.8), for a mean symptom relief of − 3.82 (SD = 3.82). In 63% of sessions, negative side effects were reported, with positive side effects reported in 97% of sessions.

**Table 1:** Session characteristics, maximum symptom relief, and side effects when using inhaled, dried *Cannabis* flower

	% or mean	*N* or Std. Dev.	Minimum	Maximum
Panel A: THC (2306 symptom-sessions, 670 users)				
%/dry wt. THC	17.27	(7.51)	0	30
THC < 10%	21%	476	0	1
THC 10–19%	39%	907	0	1
THC 20–30%	40%	923	0	1
Panel B: CBD (2306 symptom-sessions, 670 users)				
%/dry wt. CBD	6.27	(6.30)	0	29
CBD < 1%	23%	528	0	1
CBD 1–9%	45%	1049	0	1
CBD 10–30%	32%	729	0	1
Panel C: subspecies (2306 symptom-sessions, 670 users)				
Hybrid	53%	1230	0	1
*C. indica*	27%	617	0	1
*C. sativa*	20%	459	0	1
Panel D: inhalation method (2306 symptom-sessions, 670 users)				
Joint	17%	390	0	1
Pipe	40%	927	0	1
Vape	43%	989	0	1
Panel E: outcome and control variables (2306 symptom-sessions, 670 users)				
Baseline symptom intensity	5.45	(2.14)	− 10	0
Minimum symptom intensity	1.63	(1.80)	1	10
Symptom change	− 3.82	(2.16)	0	10
Panel F: side effects (1519 sessions, 559 users)				
Any negative side effect	63%	954.00	0	1
% of negative side effects	10%	(0.12)	0	0.82
Any positive side effect	97%	1471.00	0	1
% of positive side effects	28%	(0.18)	0	1
Any context-specific side effect	81%	1234.00	0	1
% of context-specific side effects	19%	(0.16)	0	0.91

Our dichotomous variables are measured {0,1} and are reported in the tables as percentages ranging from 0 to 100, along with the number of sessions reporting “1.” Our nondichotomous variables, %/dry wt. tetrahydrocannabinol (THC), %/dry wt. cannabidiol (CBD), baseline (pre-consumption) symptom intensity, minimum (post-consumption) symptom intensity, symptom change (minimum post-consumption symptom level minus starting symptom intensity), % of negative side effects, % of positive side effects, and percent of context-specific side effects, range in value as reported above with standard deviations reported in parentheses. The overall sample includes distress-related symptoms available for selection through the app: agitation/irritability, anxiety, and stress. Nineteen positive, seventeen negative, and eleven context-specific side effects were available for selection

**Fig. 1:**
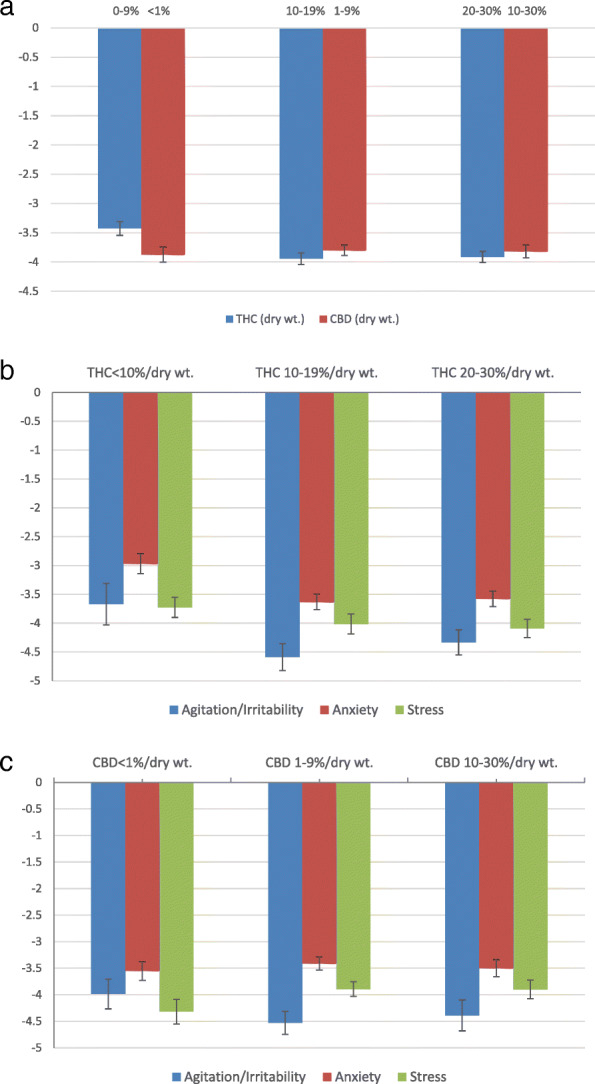
Maximum symptom relief when using inhaled, dried *Cannabis* flower by THC and CBD levels. **a** Symptom relief by CBD and THC level in overall sample. **b** Symptom relief by THC level by symptom type. **c** Symptom relief by CBD level by symptom type. Notes: Adjusted maximum symptom relief is reported, which refers to covariate-adjusted change in symptom severity (minimum symptom level reported within 4 h after session initiation minus the starting symptom level) and was obtained from a user-level fixed effects model controlling for subtype, inhalation method, and starting symptom level. Tetrahydrocannabinol (THC) and cannabidiol (CBD) are measured in %/dry wt. CBD categories are controlled for in the THC figure and THC categories are controlled for in the CBD figure. Potency levels represent percentage of labeled, laboratory-tested dried weight

Table 2 shows the effect of using inhaled, dried *Cannabis* flower on reported symptom intensity level using the fixed effects models by symptom type. On average, and as shown in Fig. 1, using an 11-point visual analog scale, respondents recorded a maximum symptom intensity reduction of 3.82 points (SE = 0.11, *p* < 0.01) in the overall distress-related symptom sample, 4.33 points in sessions treating specifically agitation/irritability (SE = 0.20, *p* < 0.01), 3.47 points in those treating anxiety (SE = 0.13, *p* < 0.01), and 3.98 points in those treating stress (SE = 0.12, *p* < 0.01).

**Table 2:** Fixed effects model for use of inhaled, dried *Cannabis* flower on reported symptom intensity level by reported symptom

	(1)	(2)	(3)	(4)
	All	Agitation/irritability	Anxiety	Stress
Post-cannabis use	− 3.824*** (0.110)	− 4.332*** (0.198)	− 3.474*** (0.125)	− 3.977*** (0.122)
Constant	5.452*** (0.055)	5.754*** (0.099)	5.246*** (0.062)	5.540*** (0.061)
Observations	4612	844	1996	1772
*R*-squared	0.644	0.707	0.635	0.686
Number of users	670	206	441	360

Each column represents a separate regression. The outcome is the reported symptom level. The explanatory variable is a dummy variable that equals to one if symptom level is reported after cannabis use and equals zero if reported before cannabis use. Models are estimated using an individual fixed effects model. Standard errors, clustered at the individual user level, are shown in parentheses

***p < 0.001

Table 3 shows the category, prevalence, and average symptom relief across each side effect ordered by frequency. Of the 47 possible side effects (17 negative, 19 positive, and 11 context-specific), the least commonly reported were the negative side effects (e.g., paranoid [4%] and experiencing a rapid pulse [3%]) and the most commonly reported were the positive side effects (e.g., relaxed [66%] and feeling peaceful [57%]), with the context-specific side effects falling in between (e.g., feeling high [47%] and thirsty [27%]). Significant changes in symptom were coreported with each of the side effect experiences.

**Table 3:** Session side effect frequencies and maximum symptom relief when using inhaled, dried *Cannabis* flower

Side effect	% session reporting	Symptom relief	Category
Relaxed	66%	− 3.96	Positive
Peaceful	57%	− 3.99	Positive
Chill	47%	− 3.83	Positive
High	47%	− 3.79	Context-specific
Comfy	41%	− 4.00	Positive
Dreamy	32%	− 3.84	Positive
Happy	29%	− 4.20	Positive
Clear	29%	− 3.80	Positive
Reflective	29%	− 4.12	Positive
Focused	28%	− 3.98	Positive
Tuned	27%	− 4.01	Positive
Light	27%	− 4.17	Positive
Thirsty	27%	− 4.13	Context-specific
Tingly	24%	− 4.16	Context-specific
Dry mouth	21%	− 4.17	Negative
Great	21%	− 4.31	Positive
Sleepy	20%	− 3.94	Context-specific
Optimistic	20%	− 4.32	Positive
Foggy	20%	− 3.80	Negative
Hungry	19%	− 3.87	Context-specific
Grateful	19%	− 4.18	Positive
Scattered	18%	− 4.19	Negative
Thinky	16%	− 3.97	Context-specific
Productive	16%	− 3.92	Positive
Couchlocked	16%	− 4.12	Context-specific
Distracted	15%	− 4.14	Context-specific
Unmotivated	14%	− 3.98	Negative
Forgetful	13%	− 4.14	Negative
Energetic	12%	− 3.95	Positive
Creative	12%	− 4.17	Positive
Restless	12%	− 4.01	Negative
Talkative	11%	− 4.09	Context-specific
Coughing	11%	− 4.08	Negative
Anxious	10%	− 3.66	Negative
Red eyes	9%	− 4.22	Negative
Frisky	9%	− 4.33	Positive
Silly	9%	− 4.10	Context-specific
Irritable	8%	− 4.23	Negative
Active	8%	− 3.98	Positive
Dizzy	8%	− 3.60	Negative
Headache	6%	− 3.71	Negative
Confused	6%	− 3.41	Negative
Paranoid	4%	− 3.09	Negative
Visuals	4%	− 4.33	Context-specific
Rapid pulse	3%	− 3.67	Negative
Clumsy	3%	− 4.21	Negative
Nausea	1%	− 3.00	Negative

Table 3 reports the percent of sessions in which the side effect was recorded as well as the average symptom relief experienced in those sessions. We categorize the side effects as negative, context-specific, or positive. Side effects were reported in 65.7% (*N* = 1519) of the 2306 sessions in the sample

Table 4 presents the results for the association between product characteristics and symptom relief in our overall sample and by symptom subgroup, treating THC and CBD levels as continuous variables. In column 1, the results show that, among product characteristics, only THC affects symptom relief—maximum symptom relief improves by 0.02 points for every one percentage point increase in THC. Comparing across columns shows that this effect is driven by sessions treating anxiety and stress rather than agitation/irritability. Longer sessions and sessions with higher starting symptoms are associated with greater symptom relief overall and across symptom types, as will be shown consistently across tables.

**Table 4:** Session product characteristics’ effects on symptom relief when using inhaled, dried *Cannabis* flower and treating cannabinoid measurements as continuous variables

	Overall Sample	Agitation/irritability	Anxiety	Stress
	(1)	(2)	(3)	(4)
THC (%/dry wt.)	− 0.020*** (0.006)	0.006 (0.018)	− 0.024** (0.008)	− 0.021* (0.009)
CBD (%/dry wt.)	− 0.002 (0.009)	0.012 (0.015)	− 0.001 (0.010)	− 0.001 (0.012)
*C. indica*	− 0.014 (0.104)	− 0.209 (0.215)	0.033 (0.133)	0.018 (0.169)
*C. sativa*	0.215 (0.111)	0.162 (0.209)	0.222 (0.127)	0.250 (0.166)
Pipe	0.093 (0.256)	0.499 (0.653)	− 0.385 (0.213)	0.498 (0.409)
Vape	0.135 (0.274)	0.708 (0.664)	− 0.222 (0.268)	0.492 (0.406)
Session length (min)	− 0.007*** (0.001)	− 0.005* (0.002)	− 0.007*** (0.001)	− 0.008*** (0.001)
Baseline symptom intensity	− 0.662*** (0.040)	− 0.694*** (0.071)	− 0.608*** (0.069)	− 0.669*** (0.052)
Constant	0.281 (0.296)	− 0.801 (0.744)	0.639 (0.379)	− 0.058 (0.437)
Number of sessions	2306	422	998	886
Number of users	670	206	441	360

Each column represents a separate regression. The outcome is the difference between the lowest reported symptom level within 4 h of initiating the session and the starting symptom level. The first column reports results for the whole sample, while columns 2 to 4 distinguish between specific distress-related symptoms reported. *C. indica* and *C. sativa* are relative to hybrid strains, and pipe and vape are relative to joint. All regressions are estimated using a fixed effects model and control for session length and baseline symptom intensity. Standard errors, clustered at the individual user level, are shown in parentheses

***p < 0.001, **p < 0.01, *p < 0.05

Table 5 uses the categorical THC and CBD measures to evaluate general nonlinearities in the effects of these variables. As shown in column 1, both ranges of THC levels above 10% are associated with greater symptom relief. In other words, THC potencies above 10% offer more relief than those below, but even higher levels of THC do not, e.g., THC levels of 20–30% offer the same amount of relief based on *F* tests of the difference between the coefficients. The results using categorical measures of THC and CBD indicate that the effect is driven by sessions treating anxiety. Small sample sizes could be a factor in the insignificance of the coefficients in column 2. As shown in column 1, the results in this table also offer suggestive evidence that strains labeled as *C. sativa* might offer less relief from distress-related symptoms than those labeled as *C. indica* or hybrid strains, but the effect is not significant in the analyses by specific symptom.

**Table 5:** Session product characteristics’ effects on symptom relief when using inhaled, dried *Cannabis* flower and treating cannabinoid measurements as categorical variables

	Overall sample	Agitation/irritability	Anxiety	Stress
	(1)	(2)	(3)	(4)
THC 10–19%/dry wt.	− 0.377** (0.123)	− 0.472 (0.469)	− 0.618*** (0.170)	− 0.024 (0.167)
THC 20–30%/dry wt.	− 0.345** (0.117)	− 0.131 (0.452)	− 0.599*** (0.165)	0.214 (0.162)
CBD 1–9%/dry wt.	0.007 (0.138)	− 0.333 (0.217)	0.120 (0.152)	0.518 (0.411)
CBD 10–30%/dry wt.	− 0.057 (0.188)	− 0.270 (0.292)	− 0.051 (0.199)	0.530 (0.409)
*C. indica*	− 0.024 (0.106)	− 0.163 (0.200)	0.037 (0.141)	− 0.110 (0.174)
*C. sativa*	0.212* (0.104)	0.068 (0.243)	0.231 (0.131)	− 0.227 (0.171)
Pipe	0.079 (0.260)	0.418 (0.646)	− 0.41 (0.220)	0.116 (0.265)
Vape	0.146 (0.274)	0.634 (0.672)	− 0.229 (0.257)	0.089 (0.313)
Session length (min)	− 0.007*** (0.001)	− 0.005* (0.002)	− 0.007*** (0.001)	− 0.008*** (0.001)
Baseline symptom intensity	− 0.661*** (0.039)	− 0.693*** (0.072)	− 0.604*** (0.067)	− 0.667*** (0.052)
Constant	0.219 (0.300)	− 0.105 (0.848)	0.649 (0.350)	− 0.402 (0.478)
Number of sessions	2306	422	998	886
Number of users	670	206	441	360

Each column represents a separate regression. The outcome is the difference between the lowest reported symptom level within 4 h of initiating the session and the starting symptom level. The first column reports results for the whole sample, while columns 2 to 4 distinguish between specific distress-related symptoms reported. The omitted category for the THC categories is THC less than 10% and for the CBD categories is CBD equal to 0%. *C. indica* and *C. sativa* are relative to hybrid strains, and pipe and vape are relative to joint. All regressions are estimated using a fixed effects model and control for session length and baseline symptom intensity. Standard errors, clustered at the individual user level, are shown in parentheses

***p < 0.001, **p < 0.01, *p < 0.05

Interactions between THC, the other product characteristics, and session length are presented in Supplemental Table 1. None of the coefficients on the interaction terms is statistically significant, implying that the effects of THC on relief from distress-related symptoms do not vary with the plant subspecies, the method of inhalation, or the session length.

Supplemental Tables 2 and 3 report the results from our robustness checks. In Supplemental Table 2, the effect of THC does not appear to vary depending on the total number of sessions entered by a user. Suggestive evidence of decreased symptom relief from strains labeled as *C. sativa* appears again in this table. Supplemental Table 3 further supports this same story. In Supplemental Table 3, our outcome variable is now the difference between the last symptom intensity reported within the specified time period and the baseline symptom intensity rather than the difference between the minimum symptom intensity reported within 4 h and the baseline symptom intensity level. Again, a one percentage point increase in THC is associated with a 0.02 point improvement in symptom relief. This table provides the strongest support for the potential that strains labeled as *C. sativa* offer less symptom relief.

Supplemental Table 4 presents our results for the effects of our independent variables on the percent of each of these side effect categories reported. None of the product characteristics or session length appears to affect negative side effect reporting. Evidence exists in columns 2 and 3 that THC increases the likelihood of reporting positive or context-specific side effects. Lastly, *C. indica* decrease the likelihood of experiencing positive side effects.

## Discussion

Feelings of distress reflect a basic dimension of human emotionality that is expressed under conditions when the individual perceives a lack of control over threatening environmental pressures and/or forces (Buchanan 2000; Gallagher et al. 2014; Vigil 2009), and it is possible that *Cannabis* usage reduces such perceptions. The current study helps explain why many patients attempting to treat feelings of distress voluntarily substitute medical cannabis for several classes of prescription medications, including those used to treat negative affect (e.g., SSRIs, SNRIs, TCAs, MAOIs, beta blockers, atypical antipsychotics, and benzodiazepines), when given the legal opportunity to do so (Bachhuber et al. 2014; Bradford and Bradford 2016; Piper et al. 2017; Powell et al. 2018; Stith et al. 2018a; Vigil et al. 2017; Wen and Hockenberry 2018). Expanding upon a previous study (Cuttler et al. 2018), our real-time effects showed that *Cannabis* flower is an effective anxiolytic medication and it is relatively fast-acting, but it can also produce negative side effects that may exacerbate momentary symptoms of negative affect in a small minority of sessions. While in some sessions users reported no change in symptom intensity levels or experiencing feelings that could contribute to distress (e.g., feeling restless), in 95% of sessions, people reported an average overall symptom intensity reduction of approximately 3.8 points on a standard 0 to 10 visual analog scale.

The current observational research design maximizes the external validity and generalizability of the findings through assessments of patients’ actual medical treatment decisions, including their choice across a range of product options, and the experienced effects of those decisions, in real-time. The mobile software technology used in the study solves the significant practical, medical, and scientific challenge of monitoring and measuring therapeutic and side effects across the vast range of products available at medical and recreational cannabis dispensaries, which vary by strains, consumption method, and major cannabinoid contents. The current results suggest benefits from patient-directed cannabis therapy as a mid-level anxiolytic treatment. Thus, despite the conventional wisdom that smoking cannabis makes one paranoid, we found consumption much more likely to be associated with relaxation and sense of calm, with users most likely to report feelings of peacefulness, optimism, and happiness. One potential explanation for the disparity between our findings and popular perceptions of cannabis is that the “paranoia” users may have historically reported could have arisen in part from cannabis’ illicit status (e.g., anxiety over committing an illegal act), rather than the plant’s typical endemic pharmacodynamic effects when consumed in contexts typical of legal medicinal use. Individual factors such as user’s experience level likely also contribute to cannabis’ effects.

This study finds that the effectiveness and side effect manifestation vary with the characteristics of the *Cannabis* flower consumed and the specific type of distress-related symptom treated. In particular, mid to higher THC levels are statistically significant predictors of increased symptom relief, while CBD levels and inhalation method (joint, pipe, vape) are largely not. In contrast, plants labeled as *C. sativa* were associated with less overall symptom relief. The relationship between higher THC and increased symptom relief appears to be driven by cannabis sessions treating specifically “anxiety” and to a lesser extent “stress” rather than “agitation/irritability,” although sample sizes are small in the subgroup analyses and some of the variation could arise from anxiety being a more clinical and clearly defined term than the other two symptoms included in our analyses.

The differences in symptom relief across THC levels might arise because THC has been shown to both decrease and increase negative mood states. However, unlike in this study, smaller doses of isolated or synthetic THC have been found to be anxiolytic and higher doses (in isolated form) appeared to be anxiogenic (Childs et al. 2017). The mechanisms by which THC potency levels can produce these biphasic effects are not fully understood and likely encompass multiple brain regions and complex interactions with other chemotypic characteristics of the plant and endogenous neurotransmitters. In rats, low doses of THC microinjected into the prefrontal cortex (e.g., 10 μg) and ventral hippocampus (e.g., 5 μg) were anxiolytic, whereas higher doses appeared to be anxiogenic. By contrast, microinjections of low doses (e.g., 1 μg) of THC in the basolateral amygdala produced anxiogenic effects, while higher doses were found to be ineffective (Rubino et al. 2008). Moreover, while low doses of THC stimulate an anxiogenic signal in the amygdala, the anxiolytic signals generated in the prefrontal cortex and hippocampus override this effect by suppressing the amygdala activation (Rubino et al. 2008).

THC’s anxiolytic effects are likely mediated by CB_1_ and CB_2_ receptors, whose respective roles appear to be to modulate neurotransmitter and cytokine release (Pertwee and Ross 2002). For example, both CB1 and 5-HT_2A_ receptors are expressed in most glutamatergic neurons in the prefrontal cortex and hippocampus (Hill et al. 2007). The CB_1_ and 5-HT_2A_ receptors have been shown to physically interact and form heteromers, and the costimulation of these CB_1_R-5-HT_2A_R heteromers appears to modulate cellular signaling in specific brain structures, including the prefrontal cortex and the hippocampus (Viñals et al. 2015). Other research suggests that anxiolytic effects of THC are mediated through the CB_1_ receptors on cortical glutamatergic terminals (Rey et al. 2012). Hence, CB_1_R-5-HT_2A_R heterodimerization may play a significant role in the reduction of glutamate levels in the prefrontal cortex and hippocampus, which could be leading the reductions in visceral feelings of distress reported by app users. However, in addition to the complexity of understanding the effects of *isolated* THC on the brain, the synergistic effects of THC and other compounds in the cannabis plant, including CBD, are even less understood. The fact that higher THC appears to confer greater anxiolytic effects in our study at *higher* THC levels suggests that the whole natural *Cannabis* plant may act very differently on the brain as compared to synthetic or derived THC isolates.

Although we were unable to account for these in our study, terpene and terpenoid contents that contribute to the overall phytocannabinoid-terpene-terpenoid synergy or “entourage effect” from whole, natural *Cannabis* plants can vary from one plant to another and by inhalation method. Many terpenes and terpenoids share a direct precursor with phytocannabinoids. For example, geranyl pyrophosphate is a precursor to the phytocannabinoids found in *Cannabis* and to the monoterpenes and monoterpenoids. Terpenes and terpenoids may comprise as much as 10% of total cannabis trichome content, and individual concentrations of > 500 ppm are considered to be of pharmacological interest. Serum terpene levels in the single-digit ng mL^−1^ range have been found to induce physiological effects potent enough to alter animal and human behavior, including anxiolytic and perceived negative side effects (Ross 2003; Russo 2011; Souto-Maior et al. 2011). For example, linalool and limonene have both demonstrated in several studies to possess potent anxiolytic properties (Carvalho-Freitas and Costa 2002; De Almeida et al. 2014; De Moraes Pultrini et al. 2006; Franco et al. 2016; Harada et al. 2018; Lima et al. 2013; Linck et al. 2010; Souto-Maior et al. 2011). β-Caryophyllene is a selective full agonist at the CB_2_ receptor and has unique effects on negative affect, making the CB_2_ receptor a prospective therapeutic target for the treatment of both anxiety and depression with cannabis (Bahi et al. 2014; Galdino et al. 2012; Kamal et al. 2018; Russo 2011). Future studies identifying strains with the most notable effects on negative affect should help elucidate why we find suggestive evidence that products labeled as “indica” and “hybrid” may be to be more anxiolytic than strains typically labeled as “sativa.” Future research will benefit from identifying and measuring the effects of particulate chemotypic profiles, including cannabinoid-terpene-flavonoid combinations, magnitudes, and ratios across varying plant strains, beyond conventional plant characteristic labeling.

The current study does have limitations, the most prominent of course being the lack of absolute experimental control (e.g., double-blinded randomization and use of a placebo intervention) and the analysis of naturalistic behaviors and dosage patterns rather than a directed and uniform regimen. Likewise, the study did not include individuals who do not use cannabis to treat their distress or any cannabis consumption sessions not tracked in the app potentially resulting in selection bias. People who choose to use cannabis to treat their distress-related symptoms may be those most likely to benefit from it or those for whom conventional treatments are less effective. The direction of the bias for app use is not as clear. Not using the app could be simply a matter of not knowing about the app or a dislike of app-based technologies, or, along with attrition, be due to dissatisfaction with cannabis or the app. Alternatively, not using the app or stopping app use could arise from satisfaction with existing cannabis use and the lack of a need to explore other product options. Within the app itself, the overt pro-cannabis language would also likely influence the type of individuals who would use the app and attract users with views aligned with the authors of the app; similar types of sample selection biases are common in large epidemiological studies where people volunteer their time to discuss or describe a discrete research topic and, in clinical trials, among people that choose to be participants in a study. Although our study extended the literature by incorporating a wider range of product characteristics than has been previously examined, we still were not able to include the full range of characteristics of products available (e.g., terpene profiles) and did not include nonflower cannabis products. We also did not account for user demographics, cannabis experience, or the concomitant use of medications other than cannabis beyond those time-invariant characteristics captured by the user fixed effects. Additional factors such as frequency of use and resultant changes in tolerance levels likely also contribute to individual differences in potential anxiogenic and anxiolytic effects, and future research may benefit by incorporating dosage and tolerance-related factors in their analyses. Finally, while improvements in testing and regulatory oversight may be reducing this issue, studies have shown that THC and CBD levels reported on product labels are often inaccurate, particularly at the higher end of the distribution, which would reduce our ability to distinguish the effects of higher versus lower potency products (Bonn-Miller et al. 2017; Vandrey et al. 2015). We attempted to mitigate this issue with our binning approach and by cutting any observations reporting THC or CBD levels exceeding 30%/dry wt. Future research would benefit from independent product testing rather than relying on user reporting based on product labels. Despite these limitations, the current finding of a dose-response effect, particularly for flower with higher THC levels, is consistent with the results of several clinical trials (Childs et al. 2017; Tambaro and Bortolato 2012) and suggests that cannabinoid contents is a major factor in *Cannabis*’ potential anxiolytic effects.

## Conclusions

In conclusion, while the clinical drawbacks of using cannabis can include the potential for dependence and addiction and increased risks of motor vehicle accidents, psychotic experiences, and short-term cognitive impairment (National Academies of Sciences, E. and M 2017; Nugent et al. 2017), the side effects reported in the current study were relatively less severe than the more serious medical and sometimes societal problems caused by some conventional prescription (e.g., benzodiazepines and barbiturates) and nonprescription (e.g., alcohol) drugs most used for treating common forms of distress (Griswold et al. 2018; Man et al. 2015; Stahre and Simon 2010). Our findings suggest that self-directed use of *Cannabis* flower, especially that with higher THC levels, is associated with significant improvements in at least short-term feelings of distress in many users, likely a contributing factor to its widespread popularity and consumption in the U.S.

## Supplementary Information

**Additional file 1: Supplemental Table 1.** Session product characteristics’ effects on symptom relief when using inhaled, dried *Cannabis* flower and interacting continuous THC and CBD measures with product characteristics and session length.

**Additional file 2: Supplemental Table 2.** Session product characteristics’ effects on symptom relief when using inhaled, dried *Cannabis* flower subsampling by the number of sessions entered by users.

**Additional file 3: Supplemental Table 3.** Session product characteristics effects on symptom relief when using inhaled, dried *Cannabis* flower by time to reported relief.

**Additional file 4: Supplemental Table 4.** Session product characteristics’ effects on side effects when using inhaled, dried *Cannabis* flower and treating cannabinoid measurements as continuous variables.

## Data Availability

Data are available from the authors upon reasonable request and with permission of MoreBetter, Ltd., the owners of the Releaf App, and the associated data. The agreement among the authors is nonexclusive and MoreBetter, Ltd. is free to enter into agreements with other researchers.

## Abbreviations

SSRI: Selective serotonin reuptake inhibitors
SNRI: Serotonin-norepinephrine reuptake inhibitors
TCA: Tricyclic antidepressant
MAOI: Monoamine oxidase inhibitors
CB_1_: Cannabinoid receptor 1
CB_2_: Cannabinoid receptor 2
