# Supplementary material for: The effectiveness of inhaled Cannabis flower for the treatment of agitation/irritability, anxiety, and common stress
Source: J Cannabis Res. 2020 Dec 9;2:47. doi: 10.1186/s42238-020-00051-z (PMC7819324; doi:10.1186/s42238-020-00051-z)
Supplement: Supplementary file 1 — Additional file 1: Supplemental Table 1. Session product characteristics’ effects on symptom relief when using inhaled, dried Cannabis flower and interacting continuous THC and CBD measures with product characteristics and session length. [file 42238_2020_51_MOESM1_ESM.docx]

Supplemental Table 1. Session product characteristics’ effects on symptom relief when using inhaled, dried *Cannabis* flower and interacting continuous THC and CBD measures with product characteristics and session length

|  | Overall Sample | Agitation/  Irritability | Anxiety | Stress |
| --- | --- | --- | --- | --- |
|  | (1) | (2) | (3) | (4) |
| THC (%/dry wt.) | -0.009 | 0.064 | -0.022 | -0.013 |
|  | (0.022) | (0.047) | (0.032) | (0.050) |
| CBD (%/dry wt.) | -0.001 | 0.014 | 0.002 | -0.003 |
|  | (0.009) | (0.017) | (0.011) | (0.012) |
| *C. indica* | -0.358 | -0.017 | -0.168 | -0.593 |
|  | (0.346) | (0.708) | (0.403) | (0.481) |
| *C. sativa* | -0.265 | -0.730 | -0.133 | 0.055 |
|  | (0.329) | (0.591) | (0.493) | (0.364) |
| Pipe | -0.057 | 1.057 | -0.444 | 0.430 |
|  | (0.436) | (0.976) | (0.572) | (1.172) |
| Vape | 0.010 | 1.252 | -0.508 | 0.563 |
|  | (0.424) | (0.853) | (0.496) | (1.193) |
| Session length (min) | -0.007*** | -0.009* | -0.007*** | -0.006** |
|  | (0.001) | (0.004) | (0.002) | (0.002) |
| THC * session length | 0.000 | 0.000 | 0.000 | -0.000 |
|  | (0.000) | (0.000) | (0.000) | (0.000) |
| THC * C. indica | -0.027 | -0.048 | -0.020 | -0.011 |
|  | (0.017) | (0.029) | (0.025) | (0.019) |
| THC * C. sativa | -0.008 | -0.054 | -0.009 | 0.024 |
|  | (0.015) | (0.030) | (0.017) | (0.018) |
| THC * Pipe | 0.009 | -0.030 | 0.005 | 0.004 |
|  | (0.020) | (0.051) | (0.031) | (0.049) |
| THC * Vape | 0.008 | -0.032 | 0.017 | -0.003 |
|  | (0.018) | (0.051) | (0.023) | (0.049) |
| Baseline Symptom Intensity | -0.663*** | -0.696*** | -0.609*** | -0.672*** |
|  | (0.039) | (0.074) | (0.068) | (0.049) |
| Constant | 0.532 | -1.002 | 0.923 | 0.014 |
|  | (0.422) | (0.924) | (0.518) | (1.109) |
| Number of sessions | 2,306 | 422 | 998 | 886 |
| Number of users | 670 | 206 | 441 | 360 |

Notes: Each column represents a separate regression. The outcome is the difference between the lowest reported symptom level within four hours of initiating the session and the starting symptom level. The first column reports results for the whole sample, while Columns 2 to 4 distinguish between specific distress-related symptoms reported. *C. indica* and *C. sativa* are relative to hybrid strains, and pipe and vape are relative to joint. All regressions are estimated using a fixed effects model and control for session length and baseline symptom intensity. Standard errors, clustered at the individual user level, are shown in parentheses. *** p<0.001, ** p<0.01, * p<0.05
