# Supplementary material for: The effectiveness of inhaled Cannabis flower for the treatment of agitation/irritability, anxiety, and common stress
Source: J Cannabis Res. 2020 Dec 9;2:47. doi: 10.1186/s42238-020-00051-z (PMC7819324; doi:10.1186/s42238-020-00051-z)
Supplement: Supplementary file 3 — Additional file 3: Supplemental Table 3. Session product characteristics effects on symptom relief when using inhaled, dried Cannabis flower by time to reported relief. [file 42238_2020_51_MOESM3_ESM.docx]

Supplemental Table 3: Session product characteristics effects on symptom relief when using inhaled, dried *Cannabis* flower by time to reported relief

|  | (1) | (2) | (3) | (4) |
| --- | --- | --- | --- | --- |
|  | 1 hour relief | 2 hours relief | 3 hours relief | 4 hours relief |
| THC (%/dry wt.) | -0.019* | -0.025*** | -0.021*** | -0.019** |
|  | (0.008) | (0.007) | (0.006) | (0.006) |
| CBD (%/dry wt.) | 0.000 | -0.006 | -0.003 | -0.002 |
|  | (0.010) | (0.009) | (0.010) | (0.009) |
| *C. indica* | 0.038 | 0.030 | -0.016 | -0.008 |
|  | (0.120) | (0.124) | (0.120) | (0.123) |
| *C. sativa* | 0.280* | 0.342** | 0.253* | 0.249* |
|  | (0.133) | (0.123) | (0.114) | (0.122) |
| Pipe | 0.039 | 0.238 | 0.203 | 0.197 |
|  | (0.295) | (0.296) | (0.290) | (0.280) |
| Vape | 0.008 | 0.168 | 0.170 | 0.185 |
|  | (0.319) | (0.310) | (0.296) | (0.281) |
| Session length (min) | 0.003* | 0.000 | -0.003* | -0.005*** |
|  | (0.001) | (0.001) | (0.001) | (0.001) |
| Baseline Symptom Intensity | -0.624*** | -0.665*** | -0.674*** | -0.672*** |
|  | (0.040) | (0.041) | (0.038) | (0.038) |
| Constant | 0.109 | 0.290 | 0.356 | 0.365 |
|  | (0.344) | (0.350) | (0.334) | (0.323) |
| Number of sessions | 2,198 | 2,266 | 2,295 | 2,306 |
| Number of users | 651 | 660 | 665 | 670 |

Notes: Each column represents a separate regression. The outcome is the difference between the last reported symptom intensity within time period specified in the column title and the baseline symptom intensity. *C. indica* and *C. sativa* are relative to hybrid strains, and pipe and vape are relative to joint. All regressions are estimated using a fixed effects model and control for session length and baseline symptom intensity. Standard errors, clustered at the individual user level, are shown in parentheses. *** p<0.001, ** p<0.01, * p<0.05
